# Effects of Prior-Task Success on Young and Older Adults’ Cognitive Performance an Evaluation of the Strategy Hypothesis

**DOI:** 10.5334/joc.17

**Published:** 2018-02-20

**Authors:** Patrick Lemaire, Fleur Brun

**Affiliations:** 1 Aix-Marseille Université, LPC and CNRS Marseille, FR

**Keywords:** effects of prior-task success, strategies, strategy selection, strategy execution arithmetic problem solving

## Abstract

In prior-task success, older adults improve cognitive performance on target tasks after successfully accomplishing a prior task. We tested the hypothesis that effects of prior-task success occur via older adults’ selecting the better strategy more often and executing strategies more efficiently on each problem under a prior-task success condition. Young and older participants accomplished computational estimation tasks (i.e., providing the best estimates to arithmetic problems) under a success or a control condition. They successfully accomplished a Stroop task or accomplished no prior task before taking the target arithmetic task. Participants had to select the better strategy on each problem in Experiment 1 and to execute a cue strategy in Experiment 2. Consistent with the strategy hypothesis, older adults, but not young adults, (a) obtained better performance, (b) used the better strategy more often, (c) inappropriately repeated the same strategy less often across successive problems, and (d) executed strategies more efficiently, under a prior-task success condition relative to a control condition. These results highlight the role of strategic variations in effects of prior-task success. They have important implications when assessing age differences in human cognition during both normal and pathological aging.

Effects of prior-task success have recently been found by Geraci and colleagues (Geraci & Miller, 2013; Geraci et al., 2016). Geraci and Miller (2013) asked young and older adults to accomplish a verbal free-recall task. Participants saw 30 randomly presented words to study for two minutes. Then, participants had to recall as many of these words as possible. Before the memory test, participants were randomly assigned to one of three experimental groups. In the first group, participants were given 30 sets of five scrambled words. For each set of five words, participants were asked to rearrange the words to form a grammatically correct four-word sentence. This task was successfully accomplished by both young and older adults (success condition). However, this sentence-scramble task was not successfully accomplished when participants had only 20 seconds (failure condition). Finally, in the control group, participants had no task prior to the target memory task. Geraci and Miller found that older adults correctly recalled more words in the success condition than in the control or failure conditions. They also found no differences between control and failure conditions in older adults, and no differences across conditions in young adults. Interestingly, effects of prior-task success led older adults to have similar memory performance relative to young adults in the success condition. Findings from additional experiments by Geraci et al. (2016) replicated these effects, but found no effects of prior-task success when the target memory task followed a motor task (i.e., throwing a bean bag into a bucket).

The present study addressed two sets of important issues regarding effects of prior-task success, one empirical, the other theoretical. Empirically, unknown is the exact nature of the relation between prior and target tasks for the effects of prior-task success to occur. Is it enough for the two tasks to be both cognitive or to involve verbal material, as in Geraci et al.’s experiments? Second, unknown is whether effects of prior-task success occur only in domains where older adults are known to undergo large cognitive declines (e.g., episodic memory) or are they found across the board of human cognition, including cognitive domains (e.g., arithmetic) where age-related declines are not as large as in memory? Third, are effects of prior-task success transient (and seen only in tasks that last two minutes, as in Geraci et al.’s experiments) or are they also seen in most cognitive tasks, usually lasting longer than two minutes? The present study addressed these issues by testing a domain-general prior task (Stroop task) and a domain-specific target (arithmetic problem solving) that participants take 30–45 minutes to accomplish.

Theoretically, to understand how effects of prior-task success occur, Geraci and colleagues (2016) tested several potential mechanisms (increased self-confidence, reduced stress and anxiety, increased positive mood, reduced age-based stereotype threat). However, they found no evidence for any of these mechanisms. Therefore, we currently ignore the mechanisms responsible for the effects of prior-task success. As a first step towards determining these mechanisms, we tested the strategy hypothesis. That is, after experiencing task success, older adults select the better strategy more often and execute available strategies more efficiently to accomplish a target task.

The strategy hypothesis tested here is based on previous findings on aging and strategic variations and on aging and strategic regulation. Numerous studies in many cognitive domains found that young and older adults differ in which (and how many) strategies they use, how often they use available strategies, and how efficient they are at selecting and executing the better strategy (see Lemaire, 2016, for a overview). For example, in arithmetic, investigated in this project, to solve two-digit addition problems, older adults tend to use fewer strategies than young adults, even if they know all the available strategies (Hodzik & Lemaire, 2011; Lemaire & Arnaud, 2008). Older adults tend to use easier strategies to say whether arithmetic inequalities like 6+3 < 11 or 271+182 < 458 are true or false (e.g., Allen et al., 1992, 1997; Duverne & Lemaire, 2004; El Yagoubi et al., 2005). Also, older adults tend to be slower to retrieve direct solutions in memory or to execute counting strategies more slowly for solving simple or complex arithmetic problems like 3 × 4 or 142 + 456 (e.g., Uittenhove & Lemaire, 2015). Finally, older adults are less systematic and less efficient at choosing the best strategy on a given arithmetic problem like when they have to select rounding strategies to find approximate products to problems like 43 × 68 (e.g., Lemaire, Arnaud, & Lecacheur, 2004). These age-related changes in strategic variations are not specific to arithmetic. They have been reported in a wide variety of cognitive domains, such as sensori-motor (e.g., Poletti et al., 2015), selective attention (McCalley, Bouwhuis, & Juola, 1995), divided attention (Maquestiaux et al., 2013), episodic memory (e.g., Froger et al., 2012; Kuhlman & Touron, 2012; Naveh-Benjamin et al., 2007), decision making (e.g., Worthy & Maddox, 2012), and spatial reasoning (Rozencwajg et al., 2005).

Research on aging and metacognition has found that spared monitoring (or evaluation of how good we are while performing a task) during aging is crucial in older adults’ strategic regulation (see Herzog & Dunlosky, 2011; Castel et al., 2016; and Hertzog, 2016, for reviews). Many studies found that older adults adjust their strategic behaviors as a function of task experience, performance self-evaluations, or affordances of task environment. For example, older adults update their monitoring after some task experience in multiple study-test experiments, allocate more re-study time on specific items that are judged less well memorized or estimated as less likely to be subsequently recalled, and on more highly valued items (e.g., Castel et al., 2012, 2013; Dunlosky & Hertzog, 1997, 2000; Hertzog et al., 2010; but see Dunlosky & Connor, 1997, for age-related differences in how participants use on-line monitoring to allocate re-study times).

How prior-task success might lead older adults to better strategy use and strategy execution can occur via several types of mechanisms, some already tested in a different cognitive context by Geraci et al. (2016), like reduced stress and anxiety, increased positive mood, or reduced age-based stereotype threat. Other potential sources include factors crucially involved in strategic variations during aging (e.g., executive control mechanisms; Hodzik & Lemaire, 2011) or other types of mechanisms like metacognitive monitoring mechanisms leading older adults to increase their level of self-confidence following task success (e.g., Hertzog, 2016).

We tested strategy selection in Experiment 1 and strategy execution in Experiment 2. In both experiments, young and older participants were asked to accomplish computational estimation tasks (i.e., providing the best estimates to addition or multiplication problems). We chose this task, because like in many other cognitive tasks, previous works showed that participants use several strategies and choose among available strategies on a problem-by-problem basis to provide estimates (e.g., LeFevre et al., 1993). Both young and older participants were tested either in a success condition (where they successfully accomplished a prior task before the arithmetic task) or in a control condition (where they accomplished no prior task). Our strategy hypothesis predicted that older adults (but not young adults) select the better strategy more often under the success condition than under the control condition (Expt. 1), and execute strategies more quickly and accurately on each problem (Expt. 2).

## Experiment 1: Does Prior-task success Change Strategy Selection?

### Method

*Participants*. A total of 92 participants were tested: 46 young and 46 older adults (see Table 1 for participants’ characteristics). Half the participants were randomly assigned to the control condition, and half to the success condition. After a presentation of the experiment, each participant signed an informed written consent, approved by the local ethic committee of Aix-Marseille University, in accordance with the ethical standards laid down in the Declaration of Helsinki.

**Table 1:** Participants’ characteristics (Expt. 1).

Characteristics	Young adults			Older adults		

	Control condition	Success condition	*F*	Control condition	Success condition	*F*

*N* (females)	23 (11)	23 (14)	—	23 (15)	23 (13)	—
Mean age (years ; months)	24;6	23;1	1.69	71;0	70;0	0.26
Age range	18;0–32;0	18;0–30;0	—	59;2–84;4	61;0–83;0	—
Number of years of education	11.5	11.7	0.2	11.9	11.8	.14
Arithmetic fluency (SD)	59.7 (12.1)	60.0 (15.9)	0.00	69.6 (9.0)	70.7 (13.1)	0.12
MHVS^1^ (SD)	24.4 (4.8)	23.2 (4.8)	0.74	24.0 (2.3)	24.5 (4.6)	0.58
MMSE^2^	—	—	—	29.0	29.1	—

*Note*. ^1^Mill-Hill Vocabulary Scale, ^2^Mini-Mental State Examination.

*Stimuli for the Stroop task*. Stimuli were 40 color words typewritten on a sheet of paper (display of 8 lines, 5 columns). Words were *red, blue, yellow*, or *green*. The ink color in which words were written could be red, blue, yellow, or green. The color of the word was always different from its meaning (i.e. the word *green* was presented in blue ink).

*Stimuli for the arithmetic task*. Each of the 32 trials consisted of three consecutive two-digit addition problems. Each trial was followed by a series of four letters to avoid carry-over effects from one trial to the next (e.g., participants may repeat the same strategy on the last problem of a given trial and the first problem of the next trial independently of which strategy is the best on each of these problems). Half the four-letter series included either only consonants or only vowels, and half included both types of letters. To examine if prior-task success changes participants’ tendency to repeat the same strategy across problems, each series of three addition problems were composed of two prime problems followed by a target problem (e.g., 48 + 56, 29 + 76, 68 + 34, with 48 + 56 as the first prime problem, 29 + 76 as the second prime problem, and 68 + 34 as the target problem). Prime and target problems were selected so that they had correct sums of comparable magnitudes when solved with each rounding strategy. More precisely, mean correct sums were 101 (range: 64—136) for prime problems and 98 (range: 68—129) for target problems, *F* < 1. The prime-target structure of trials followed several previous studies of ours (Hinault et al., 2017; Hinault, Lemaire, & Phillips, 2016; Lemaire & Leclère, 2014), as findings from these studies (e.g., participants’ better strategy choices on prime and target problems) revealed that this structure was appropriate to investigate age-related differences in strategy use and strategy execution. Also, this prime-target structure was used here to test whether prior-task success changes (a) percentages of better strategy use and strategy performance on prime problems for which better strategy is easier to determine and on target problems for which better strategy is hard to select, and (b) how often participants tend to repeat the same strategy across prime and target problems.

Half the prime problems had their unit digits of both operands smaller than 5 (e.g., 42 + 51) so that participants would obtain better estimates (i.e., sums closer to correct sums) on these rounding-down problems when correctly executing the rounding-down strategy. The other prime problems had their unit digits of both operands larger than 5 (e.g., 38 + 56) so that the rounding-up strategy was the better strategy for these rounding-up problems. Prime rounding-down and rounding-up problems had comparable mean sums and mean percent deviations between best estimates and correct sums.

All target problems included one operand with its unit digit smaller than 5 and the other with its unit digit larger than 5. These prime and target problems were selected because previous studies (e.g., Lemaire et al., 2014) showed that participants almost systematically select the rounding-down strategy on rounding-down problems and the rounding-up strategy on rounding-up problems but have more difficulties in selecting the better strategy on target problems. It is harder to select the better strategy on target problems, because the size of unit digits suggests that either the rounding-down or the rounding-up strategy is the best. In contrast, the size of unit digits clearly and unambiguously indicate that prime problems with both unit digits smaller than 5 are best estimated with the rounding-down strategy and problems with both unit digits larger than 5 are best estimated with the rounding-up strategy. Moreover, there were two types of target problems, neutral and non-neutral problems. Neutral problems had the sum of their unit digits equal to 10 (e.g., 68 + 32) so that no strategy was the better strategy for these problems. Half of the non-neutral problems had the sum of their unit digits smaller than 10 (e.g., 68 + 31), so that rounding down was the better strategy, and the other non-neutral target problems had the sum of their unit digits larger than 10 (e.g., 68 + 34), so that rounding up was the better strategy. Correct sums and mean percent deviations for each type of problems were controlled so that strategy selection would not be contaminated by these factors.

Finally, following previous findings in arithmetic (see Cohen-Kadosh & Dowker, 2015, for an overview), we controlled the following factors: (a) no operands had a 0 unit digit (e.g., 20 + 63), or a 5 unit digit (e.g., 25 + 63); (b) no digits were repeated within operands (e.g., 22 + 63); (c) the first operand was larger than the second operand in half the problems, and vice versa; (d) no operand had its closest decade equal to 0, 10, or 100; and (e) rounded operands were never the same across two successive problems in a given trial (e.g., if one problem in a trial was 32 + 64, the next problem could not be 31 + 62).

*Procedure*. Participants were individually tested in one session. First, older adults took the Mini-Mental State Examination, MMSE (Folstein, Folstein, & McHugh, 1975) to exclude participants with potential Alzheimer’s disease. All older participants had scores larger than 27; therefore, none were excluded. Then, both young and older adults took the Mill-Hill Vocabulary test (Raven, Court, & Raven, 1986) and an independent pencil-and-paper arithmetic test (French Kit; French et al., 1963) to assess participants’ verbal and arithmetic fluency.

Participants in the success condition were given the prior Stroop interference task. They had to name the color of each word, without paying attention to the word itself. They were instructed to read words, line by line. As they had a maximum of three minutes, participants performed the task perfectly. Young and older adults took 41 sec. and 60 sec., respectively to read the 40 words (both groups made an average of two errors which they corrected as they were instructed to correct possible errors). They were told their performance, and we congratulated them for their speed. A pencil-and-paper version of the Stroop task was tested so that participants in the success condition would not receive a warm up on the task apparatus. Participants in the control condition did not complete the prior task. We did not test a failure condition, because Geraci et al. (2013, 2016) found no differences between control and failure conditions, and because the critical comparisons here were between control and success conditions.

Next, all participants completed the computational estimation task. The two-digit addition problems were presented horizontally in a 84 point Bold Courier font (black colour) in the middle of a 14-inch computer screen. The software (E-Prime) controlled stimulus display and latency collection.

Participants were told that they were going to do a computational estimation task. The computational estimation task was explained as giving an approximate answer to an arithmetic problem that is as close as possible to the correct answer without actually calculating the correct answer. Thus, participants had to estimate the sum of the problem displayed on the screen, trying to choose the best rounding strategy. They were told to use only two rounding strategies, the rounding-down strategy (e.g., rounding both operands down to the nearest decades, for instance doing 60 + 40 to estimate 63 + 48) or the rounding-up strategy (e.g., rounding both operands up to the nearest decades, for instance doing 70 + 50 to estimate 63 + 48).

After an initial practice period including 12 problems (six with each rounding strategy), all participants had no difficulties with either rounding strategy. Then, participants practiced for eight trials (each involving three addition problems and a series of four letters) for them to get familiarized with the procedure and the structure of each trial. Finally, in the experimental part, they saw 32 trials (i.e., 96 addition problems and 32 series of letters) with a break in-between each block of 16 trials.

Each trial started with a 300-ms blank screen before a 400-ms fixation cross displayed at the centre of the screen, followed by the first prime problem, another 300-ms blank screen, a 400-ms fixation cross, and the second prime problem. A blank screen followed participants’ responses to the second problem for 300 ms. Then, the 400-ms fixation cross and the target problem were displayed. The timing of each response began when the problem appeared on the screen and ended when the experimenter pressed the space bar of the computer keyboard, the latter event occurring as soon as possible after participants’ responses. Participants were asked to calculate out loud so that we could be sure of which strategy they used. On each problem, the experimenter recorded participants’ responses and strategy choices.

After the target problem of each trial, a blank screen followed participant’s response for 300 ms. Then, the warning signal appeared for 400 ms followed by four letters. Letters were displayed until the participant responded, pressing on the “L” key of the AZERTY keyboard when the four letters were only consonants (e.g., *trlc*) or only vowels (e.g., *aeio*), and on the “S” key when the four letters included both consonants and vowels (e.g., *ubqi*). A blank screen was finally displayed for 1000 ms at the end of each trial and before the next trial started. Each session lasted approximately 30–45 minutes.

### Results

Results are reported in two main parts. We analysed effects of prior-task success on better strategy selection and on strategy performance for prime problems first and then for target problems. In all results, unless otherwise noted, differences are significant to at least *p* < .05.

#### Effects of Prior-task success on Prime Problems

Better strategy selection and strategy performance (mean correct estimation times and percentages of errors) on prime and target problems (Tables 2 and 3) were analyzed with mixed-design ANOVAs, 2 (Age: young, older adults) × 2 (Condition: control, success) × 2 (Problem Type: rounding-down, rounding-up), with repeated measures on the last factor.

**Table 2:** Mean percentages of better strategy selection, mean estimation times, and percentages of errors in young and older adults under the success and control conditions for rounding-down and rounding-up prime problems (Expt. 1).

Problem Type	Young adults				Older adults				*Means*			

	Control	Success	*Differences*	*Means*	Control	Success	*Differences*	*Means*	Control	Success	*Differences*	*Means*

*Percentages of Better Strategy Selection*												

Rounding-Down	96.2	97.8	–1.6	97.0	97.8	98.1	–0.3	98.0	97.0	98.0	–1.0	97.5
Rounding-Up	98.4	94.8	3.5	96.6	92.7	93.8	–1.1	93.2	95.5	94.3	1.2	94.9
*Means*	97.3	96.3	1.0	96.8	95.2	95.9	–0.7	95.6	96.3	96.1	0.1	96.2
*Estimation Times (in ms)*												

Rounding-Down	3424	3748	–325	3586	5193	4037	1156	4615	4308	3893	415	4100
Rounding-Up	3758	4227	–469	3993	5780	4344	1436	5062	4769	4286	483	4528
*Means*	3591	3988	–397	3789	5486	4191	1296	4838	4539	4089	449	4314
*Percentages of errors*												

Rounding-Down	1.1	0.0	1.1	0.5	0.6	0.0	0.6	0.3	0.8	0.0	0.8	0.4
Rounding-Up	1.4	0.1	1.3	0.8	1.1	0.4	0.6	0.8	1.2	0.3	0.9	0.8
*Means*	1.3	0.1	1.2	0.7	0.8	0.2	0.6	0.5	1.0	0.1	0.9	0.6

**Table 3:** Mean percentages of better strategy selection, mean estimation times, and percentages of errors in young and older adults in the success and control conditions for rounding-down, rounding-up, and neutral target problems (Expt. 1).

Problem Type	Young adults				Older adults				*Means*			

	Control	Success	*Differences*	*Means*	Control	Success	*Differences*	*Means*	Control	Success	*Differences*	*Means*

*Percentages of Better Strategy Selection*												

Rounding-Down	70.4	72.1	1.7	71.2	64.3	72.3	8.0	68.3	67.3	72.2	4.8	69.8
Rounding-Up	78.4	72.3	–6.1	75.3	65.7	80.6	14.8	73.1	72.0	76.4	4.4	74.2
*Means*	74.4	72.2	–2.2	73.3	65.0	76.4	11.4	70.7	69.7	74.3	4.6	72.0
*Estimation Times (in ms)*												

Rounding-Down	4526	4679	153	4602	6287	4950	–1338	5619	5406	4814	–592	5110
Rounding-Up	4645	4891	246	4768	6736	5240	–1496	5988	5690	5066	–625	5378
Neutral	4375	4504	129	4440	6208	4898	–1310	5553	5292	4701	–590	4996
*Means*	4515	4691	176	4603	6410	5029	–1381	5720	5463	4860	–603	5162
*Percentages of errors*												

Rounding-Down	0.5	1.7	1.2	1.1	1.8	0.6	–1.2	1.2	1.2	1.2	0.0	1.2
Rounding-Up	0.5	1.2	0.6	0.9	2.0	0.0	–2.0	1.0	1.3	0.6	–0.7	0.9
Neutral	2.4	1.5	–0.8	1.9	4.1	0.9	–3.2	2.5	3.2	1.2	–2.0	2.2
*Means*	1.1	1.5	0.3	1.3	2.6	0.5	–2.1	1.6	1.9	1.0	–0.9	1.4

*Better strategy selection on prime problems*. If participants used the better strategy on both prime problems within one trial, better strategy selection was coded 1; otherwise, it was coded 0. All participants selected the better strategy more often on rounding-down problems (97.5%) than on rounding-up problems (94.9%; *F*(1,88) = 5.06, *MS*e = 60.6, \eta _p^{\it 2} = .05). The marginally significant Age × Problem Type interaction (*F*(1,88) = 3.59, *MS*e = 60.6, \eta _p^{\it 2} = .04, *p* = .06) revealed that this problem type effect occurred in older adults (98.0% vs. 93.2%; *F*(1,44) = 5.44, *MS*e = 26.4, \eta _p^{\it 2} = .11) but not in young adults (97.0% vs. 96.6%; *F* < 1). No other effects came out significant (*Fs* < 2.0).

*Strategy performance on prime problems*. Young adults (3789 ms) were faster than older adults (4838 ms; *F*(1,88) = 12.12, *MS*e = 4177552.9, \eta _p^{\it 2} = .12). Moreover, all participants executed the rounding-down strategy (4100 ms) more quickly than the rounding-up strategy (4528 ms; *F*(1,88) = 38.67, *MS*e = 217040.4, \eta _p^{\it 2} = .31). The main effect of condition was not significant (*F* = 2.22), but the Age × Condition interaction (*F*(1,88) = 7.89, *MS*e = 4177552.9, \eta _p^{\it 2} = .08) showed that the difference between control and success conditions occurred in older adults only. They were faster in the success (4191 ms) than in the control condition (5486 ms; *F*(1,44) = 6.16, *MS*e = 6271702.8, \eta _p^{\it 2} = .12). Comparing young and older adults within each condition showed that young adults were faster than older adults in the control condition only (*F*(1,44) = 16.66, *MS*e = 4960259, \eta _p^{\it 2} = .27).

All participants made fewer errors in the success (0.1%) than in the control condition (1.0%; *F*(1,88) = 5.41, *MS*e = 6.8, \eta _p^{\it 2} = .06). They tended to err less on rounding-down problems (0.4%) than on rounding-up problems (0.8%; *F*(1,88) = 3.49, *MS*e = 1.6, \eta _p^{\it 2} = .04, *p* = .06).

#### Effects of Prior-task success on Target Problems

**Figure 1:**
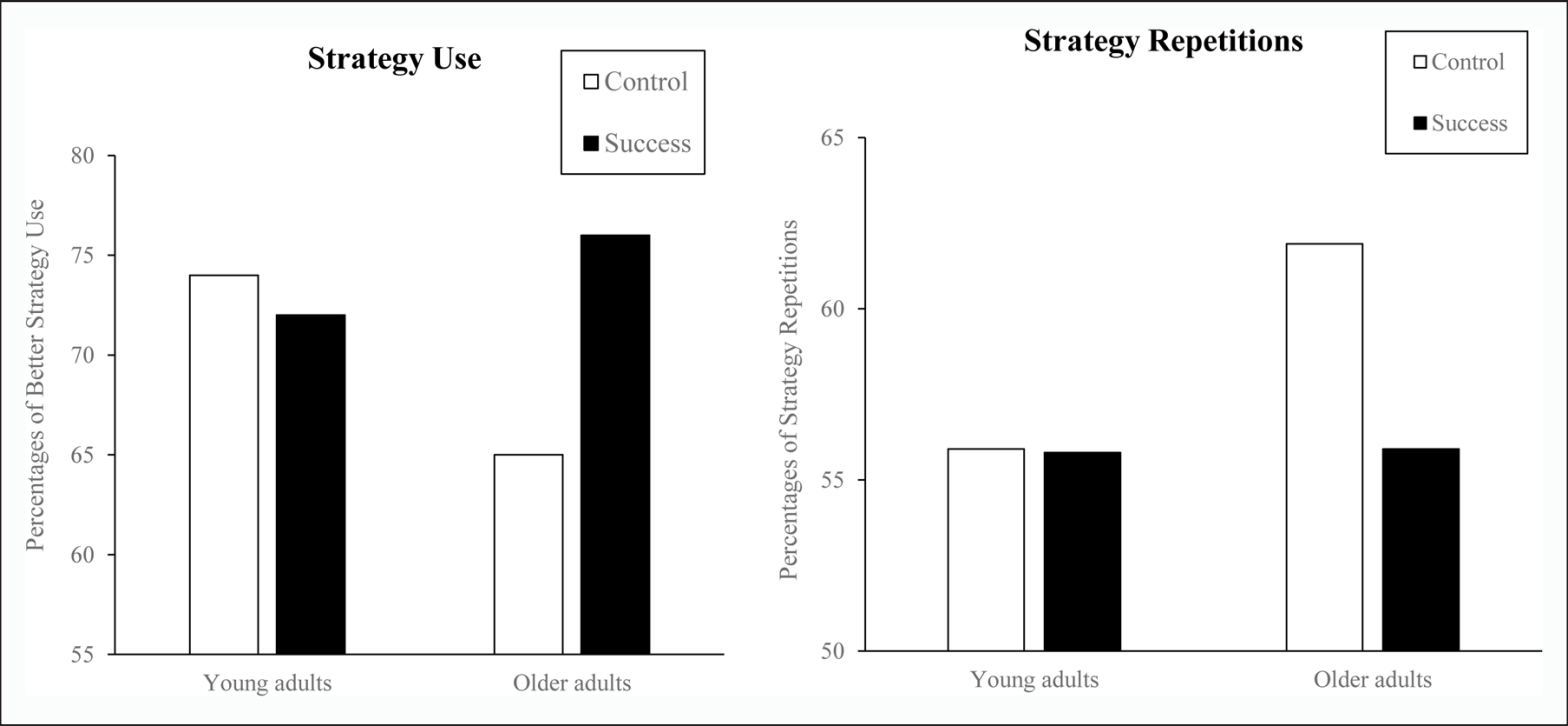
Young and Older Adults’ Strategy Use and Strategy Repetitions Under Control and Success Conditions (Expt. 1).

*Better strategy selection on target problems*. The Age × Condition interaction (*F*(1,88) = 4.30, *MS*e = 495.2, \eta _p^{\it 2} = .05) revealed that older adults selected the better strategy more often in the success than in the control condition (76.4% vs. 65.0%; *F*(1,44) = 6.06, *MS*e = 493.5, \eta _p^{\it 2} = .12). Young adults selected the better strategy equally often in the success and control conditions (72.2% vs. 74.4%; *F* < 1). They selected the better strategy more often than older adults in the control condition (*F*(1,44) = 4.79, *MS*e = 534, \eta _p^{\it 2} = .08). The age effect did not reach significance (*F* < 1) in the success condition (see Figure 1, left panel).

*Strategy performance on target problems*. Young adults (4603 ms) were faster on target problems than older adults (5720 ms; *F*(1,88) = 10.88, *MS*e = 7902400.8, \eta _p^{\it 2} = .11). The main effect of prior-task success was marginally significant (*F*(1,88) = 3.17, *MS*e = 7902400.8, \eta _p^{\it 2} = .03, *p* = .08). Most importantly, the Age × Condition interaction (*F*(1,88) = 5.29, *MS*e = 7902400.8, \eta _p^{\it 2} = .06) revealed that older adults were faster in the success than in the control condition (5029 vs. 6410 ms; *F*(1,44) = 6.19, *MS*e = 10627505.7, \eta _p^{\it 2} = .12) and that young adults were equally fast in each condition (4691 vs. 4515 ms; *F* < 1). Moreover, there was a main effect of problem type (*F*(2,176) = 7.03, *MS*e = 502076.5, \eta _p^{\it 2} = .04). All participants were slower on rounding-up problems (5378 ms) than on rounding-down problems (*F*(1,88) = 7.95, *MS*e = 414449.7, \eta _p^{\it 2} = .08) or than on neutral problems (*F*(1,88) = 10.94, *MS*e = 612072.2, \eta _p^{\it 2} = .11); and they solved neutral and rounding-down problems (4996 ms vs. 5110 ms; *F* = 1.24) equally fast. Also, young adults were faster than older adults in the control condition (*F*(1,44) = 13.32, *MS*e = 9301399, \eta _p^{\it 2} = .23), but not in the success condition (*F* < 1).

The Age × Condition interaction (*F*(1,88) = 4.69, *MS*e = 22.0, \eta _p^{\it 2} = .05) was significant on percentages of errors, as only older adults made fewer errors in the success than in the control condition (0.5 vs. 2.6%; *F*(1,44) = 6.20, *MS*e = 25.3, \eta _p^{\it 2} = .12).

*Strategy perseverations on target problems*. In the final analysis, we aimed at determining whether prior-task success changed participants’ tendency to repeat the same strategy across prime and target problems, especially when it is not appropriate (i.e., unrepeated problems). An ANOVA was performed on mean percentages of strategy repetitions (i.e., if participants used the same strategy on prime and target problems, strategy repetition was coded 1; otherwise, it was coded 0; see means in Table 4) with a mixed design: 2 (Age: young, older adults) × 2 (Condition: control, success) × 3 (Target type: neutral, repeated, unrepeated), with repeated measures on the last factor.

**Table 4:** Mean percentages of strategy repetitions in young and older adults under the success and control conditions for neutral, repeated, and unrepeated trials (Expt 1).

Trials	Young adults				Older adults			

	Control	Success	*Differences*	*Means*	Control	Success	*Differences*	*Means*

Neutral	59.5	58.8	0.6	59.1	62.6	60.3	2.3	61.5
Repeated	78.3	77.9	0.4	78.1	76.8	80.1	–3.3	78.5
Unrepeated	30.0	30.8	–0.7	30.4	46.3	27.3	19.0	36.8
*Means*	55.9	55.8	0.1	55.9	61.9	55.9	6.0	58.9

*Note*. Differences: Control – Success.

The effect of target type, *F*(2,176) = 150.48, *MS*e = 309.2, \eta _p^{\it 2} = .46, showed that all participants repeated the same strategy more often on repeated (78.3%) than on neutral (60.3%; *F*(1,88) = 71.20, *MS*e = 208.7, \eta _p^{\it 2} = .45) or than on unrepeated targets (33.6%; *F*(1,88) = 181.65, *MS*e = 505.8, \eta _p^{\it 2} = .67), and more often on neutral than on unrepeated targets (*F*(1,88) = 154.15, *MS*e = 213.0, \eta _p^{\it 2} = .64). The Age × Condition × Target type was marginally significant, *F*(2,176) = 2.82, *MS*e = 309.2, \eta _p^{\it 2} = .02, *p* = .06.

**Figure 2:**
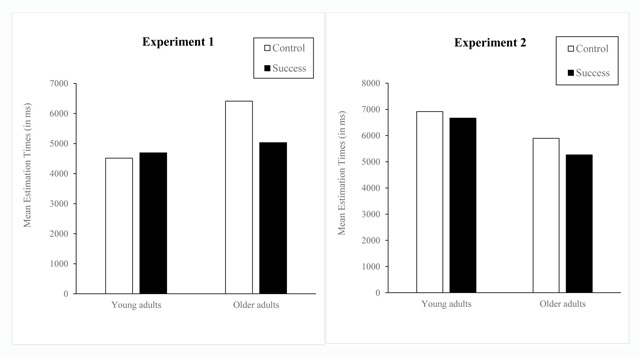
Mean Estimation Performance in Young and Older Adults Under Control and Success Conditions (Expts. 1 and 2).

Separate analyses in each age group revealed that there were significant effects of problem types in both young and older adults (*F*s > 62.35). Moreover, young adults tended to repeat the same strategy equally often in the control and success conditions (55.9% and 55.8%; *F* < 1). Interestingly, older adults repeated the same strategy less often in success than in control condition (55.9% vs. 61.9%; *F*(1,44) = 4.02, *MS*e = 308.0, \eta _p^{\it 2} = .08, *p* = .05; see Figure 2, right panel), especially while they solved unrepeated targets, as seen in the significant Condition × Target type interaction (*F*(2,88) = 4.80, *MS*e = 324.0, \eta _p^{\it 2} = .05). This is because older adults repeated the same strategy less often in the success than in the control condition only on unrepeated targets (46.3% vs. 27.3%; *F*(1,44) = 11.43, *MS*e = 363.8, \eta _p^{\it 2} = .21), but not on neutral or repeated targets (*F*s < 1). Young adults repeated the same strategy less often than older adults in the control condition (*F*(1,44) = 7.14, *MS*e = 425.8, \eta _p^{\it 2} = .14). The age effect did not reach significance in the success condition (*F* < 1).

In sum, Experiment 1 replicated effects of prior-task success originally found by Geraci and colleagues (2013, 2016). Older adults (but not young adults) obtained better performance in a target task after experiencing success in a different cognitive task. Geraci et al. had found effects of prior-task in a episodic memory task as a target task following a prior sentence-scramble task. The present findings generalize these effects to another cognitive domain, arithmetic, after experiencing success in an unrelated, cognitive Stroop task. Finally, these findings show that effects of prior-task success are not transient and do not occur in cognitive tasks that last only two minutes. They are also found in tasks that participants take 30–45 minutes to accomplish.

Most importantly, results of Experiment 1 are consistent with the hypothesis that effects of prior-task success occur via changes in strategy use and strategy execution. To confirm that participants executed strategies more quickly in the success condition, Experiment 2 controlled for strategy selection by asking participants to execute pre-selected strategies.

## Experiment 2: Does Prior-Task Success Changes Strategy Execution?

### Method

A total of 114 participants were tested: 54 young adults and 60 older adults (see Table 5 for participants’ characteristics), half of whom were randomly assigned to the control condition, and half to the success condition.

**Table 5:** Participants’ characteristics (Expt. 2).

Characteristics	Young adults			Older adults		

	Control condition	Success condition	*F*	Control condition	Success condition	*F*

*N* (females)	27 (23)	27 (21)	—	30 19)	30 (17)	—
Mean age (years;months)	20;4	20;1	0.12	71;5	73;9	2.43
Age range	17;10–34;9	18;4–27;9	—	58;2–85;2	65;1–85;1	—
Number of years of education	11.8	11.6	0.91	11.7	11.5	0.73
Arithmetic fluency	39.2	39,9	0.05	76.0	77.3	.06
MHVS^1^	18.4	19.0	0.10	24.3	25.9	1.57
MMSE^2^	—	—	—	29.7	29.5	—

*Note*. ^1^ Mill-Hill Vocabulary Scale, ^2^ Mini-Mental State Examination.

*Stimuli*. The same stimuli and procedure as in Experiment 1 were used for the Stroop prior task. The Computational Estimation task included 64 two-digit multiplication problems presented in a standard form (e.g., 21 × 58). Half the problems (*N* = 32) included homogeneous unit digits, the other problems included heterogeneous unit digits. The unit digit of both operands was smaller than 5 (e.g., 32 × 63) in half the homogeneous problems and larger (e.g., 38 × 69) than five in the other homogeneous problems. In problems with heterogeneous unit digits, the unit digit of one operand was smaller than 5, and that of the other operand was larger than 5 (e.g., 42 × 69). Homogeneous and heterogeneous problems had comparable exact products. Also, half the homogeneous and heterogeneous problems were best estimated (i.e., closest products from correct products) with the rounding-down strategy (e.g., 56 × 32; rounding-down problems). The other problems were best estimated with the rounding-up strategy (e.g., 24 × 49; rounding-up problems). The same factors as in Experiment 1 were controlled in problem selection (e.g., side of the larger operand, size of correct products).

*Procedure*. Like in Experiment 1, participants were individually tested in one session and we first assessed participants’ verbal and arithmetic fluencies with the Mill-Hill Vocabulary and the French kit tests, respectively. Older adults also took the MMSE. Young and older adults tested in the success condition took 40 sec. and 59 sec., respectively to read the 40 interference words in the prior Stroop task (both groups made an average of three errors which they corrected as they were instructed to correct possible errors).

Next, all participants completed the computational estimation task as in Experiment 1, except that they did not have to select strategies. To assess strategy execution independently of strategy choice demands and of potential interference between required and best strategy, participants saw a strategy cue above each problem (i.e., « RD », or « RU »), and the cued strategy was always the better strategy (i.e., the strategy that yielded the closest answer from the correct product).

### Results

Mean correct estimation latencies and percentages of errors (Table 6; Figure 2, right panel) were analyzed with mixed-design ANOVAs, 2 (Age: young, older adults) × 2 (Condition: control, success) × 2 (Strategy: rounding-down, rounding-up), with repeated measures on the last factor.^1^

**Table 6:** Mean estimation times and percentages of errors in young and older adults under the success and control conditions for the rounding-down and rounding-up strategies (Expt. 2).

Conditions	Young adults			Older adults			*Means*		

	RD	RU	*Means*	RD	RU	*Means*	RD	RU	*Means*

*Estimation Times (in ms)*									

Control	6264	7566	6915	4594	5149	4871	5428	6357	5893
Success	6148	7184	6666	3581	4151	3866	4865	5667	5266
*Means*	6206	7375	6571	4088	4650	4369	5147	6012	5580
*Differences*	116	382	249	1013	998	1006	564	690	627
*Percentages of Errors*									

Control	7.8	14.0	10.9	3.3	7.2	5.3	5.6	10.6	8.1
Success	6.0	13.4	9.7	1.5	2.1	1.8	3.7	7.8	5.8
*Means*	6.9	13.7	10.3	2.4	5.1	3.6	4.7	9.2	6.9
*Differences*	1.9	0.6	1.2	1.8	4.7	3.4	1.8	2.8	2.3

*Note*. RD: Rounding-down strategy; RU: Rounding-up strategy. Differences = Control – Success.

Older adults (5369 ms) were faster than young adults (6791 ms; *F*(1,110) = 70.08, *MS*e = 4758159.5, \eta _p^{\it 2} = .39). All participants executed the rounding-down strategy (5147 ms) more quickly than the rounding-up strategy (6012 ms; *F*(1,110) = 193.52, *MS*e = 220003.5, \eta _p^{\it 2} = .64). Also, the Age × Strategy interaction (*F*(1,110) = 23.79, *MS*e = 220003.5, \eta _p^{\it 2} = .18) revealed that this strategy effect was larger in young adults (1169 ms; *F*(1,52) = 131.91, *MS*e = 279649.6, \eta _p^{\it 2} = .72) than in older adults (562 ms; F(1,52) = 56.90, MSe = 166527.6, \eta _p^{\it 2} = .50). Most importantly, the Age × Condition interaction was significant, *F*(1,110) = 7.71, *MS*e = 4758159, \eta _p^{\it 2} = .05. Older adults were faster in the success condition than in the control condition (3866 ms vs. 4871 ms; *F*(1,58) = 9.32, *MS*e = 325604, \eta _p^{\it 2} = .14) whereas young adults were not significantly faster in the success than in the control conditions (*F* < 1). Although older adults were faster than young adults in both the control (*F*(1,55) = 18.12, *MS*e = 6551774, \eta _p^{\it 2} = .25) and success conditions (*F*(1,55) = 75.19, *MS*e = 2964544, \eta _p^{\it 2} = .58), age-related differences increased from the control (+2044 ms) to the success condition (+2800 ms). This increase resulted from older adults’ increased speed under the success condition relative to the control condition.

Older adults made fewer errors than young adults (3.6% vs. 10.3%, respectively; *F*(1,110) = 22.72, *MS*e = 113.1, \eta _p^{\it 2} = .17). Participants erred less with the rounding-down strategy (4.7%) than with the rounding-up strategy (9.2%; *F*(110) = 38.96, *MS*e = 30.1, \eta _p^{\it 2} = .26). The Age × Strategy interaction (*F*(1,110) = 9.89, *MS*e = 30.1, \eta _p^{\it 2} = .08) showed that this strategy effect occurred in young adults (*F*(1,52) = 34.53, *MS*e = 36.4, \eta _p^{\it 2} = .40) but not in older adults (*F* = 2.49, *ns*). Although the Age × Condition was not significant (*F* < 1), we found that older adults made more errors in the control (5.3%) than in the success condition (1.8%; *F*(1,52) = 4.25, *MS*e = 83.9, \eta _p^{\it 2} = .07) and that young adults erred equally often in the success and control conditions (9.7% vs. 10.9%; *F* < 1). No other effects came out significant on estimation times and percent errors (*Fs* < 2.0, *ns*).

In sum, Experiment 2 found that, relative to a control condition, older (but not young) participants were faster and more accurate under a success condition, even when they do not select strategies. It was interesting that we did find effects of prior-task success in older adults while older adults had higher arithmetic fluency scores. The reason for why this is important is that it discards the hypothesis that effects of prior-task success occurred in previous works on memory because older adults have lower memory capacities. As older adults in this experiment had higher arithmetic fluency, effects of prior-task success cannot be the result of group-related differences in baseline task or domain performance.

### General Discussion

We found effects of prior-task success while participants accomplished arithmetic problem solving tasks. Older adults, but not young adults, obtained better performance on a target computational estimation task after experiencing success in a prior Stroop task. In Experiment 1, older adults were 1295 ms (corresponding Cohen’s *d* = .748) faster in the success condition than in the control condition on prime problems, and 1381 ms (corresponding Cohen’s *d* = .75) on target problems. In Experiment 2, older adults were 1005 ms faster in the success than in the control condition (corresponding Cohen’s *d* = .80). Effects of prior-task success occurred via older adults’ selecting the better strategy more often and executing strategies more efficiently on each problem under success condition. Because a strategy can be defined as “a procedure or set of procedures for achieving a higher level goal or tasks” (Lemaire & Reder, 1999, p. 365), older adults changed the set of mechanisms they use to accomplish the target task after successfully accomplishing a prior task. They also changed the way they execute the same mechanisms when they did not use different mechanisms. These findings have important implications for understanding how prior-task success improves older adults’ cognitive performance.

Our findings suggest that effects of prior-task success are not limited to cognitive domains in which aging has large negative effects, like memory where they were originally found by Geraci and her colleagues (2013, 2016). These effects are also seen in domains where young and older adults have comparable baseline performance and even when older adults have better performance than young adults (e.g., arithmetic). Also, effects of prior-task success are apparently not limited to short target tasks. They also occurred in the present arithmetic tasks that participants needed 30–45 minutes to accomplish. Thus, these success effects are not transient, but are robust, reliable, and can be rather long lasting.

The present findings that effects of prior-task success occur via strategic variations were obtained in the arithmetic domains where strategic variations crucially influence participants’ performance and age-related changes in participants’ performance (see Lemaire, 2016, for an overview). Future studies may collect further evidence that these findings are not limited to the arithmetic domain, but also hold to several cognitive domains, including the memory domain where effects of prior-task success were first found by Geraci and colleagues (2013, 2016). Because strategic variations have been found in a wide variety of cognitive domains, the present study suggests that wherever they may occurr, effects of prior-task success may occur via older adults’ using different strategy under success condition, executing existing strategies more efficiently, or via both better strategy selection and more efficient strategy execution.

It could be argued that effects of prior-task success are only warm-up effects. Indeed, the success group received warm-up with a cognitive task whereas the control group had no warm-up whatsoever. However, we believe that this is not the case for two reasons. First, Gearci and colleagues (2013, 2016) tested failure conditions where participants had the same prior tasks as in the success condition, but were time-limited. Thus, participants in the failure condition received a cognitive warm-up prior to the target task. Yet, they obtained similar performance as participants in the control condition. Second, in the present experiments, participants of both the control and success conditions had equal opportunities to warm up as they practiced the strategies and target tasks first on 12 problems then on eight trials (each with three problems) before the target task. These suggest that experiencing success with prior tasks is key to effects of prior-task success.

Unknown is how prior-task success impacts strategy selection and execution. Several mechanisms can be tested in future research. First, previous works suggest that mechanisms like executive control, crucially involved in better strategy selection (Hodzik & Lemaire, 2011), may be influenced by prior-task success. Second, mechanisms like reduced stress and anxiety, or increased positive mood might moderate effects of prior-task success. Third, prior-task success may reduce stereotype activation, which may influence older adults’ confidence and executive functioning, as well as older adults’ regulatory focus (e.g., Barber & Mather, 2014, and Lamont, Swift, & Abrams, 2015, for reviews) or how older adults approach cognitive tasks. Although Geraci et al. (2016) did not find evidence for these mechanisms in their study of memory, future studies may test these mechanisms in the context of arithmetic.

Other potential mechanisms concern metacognitive mechanisms. Previous studies found that older adults are good at monitoring their cognitive performance and that such monitoring guides strategic regulation (see Castel et al., 2016; Hertzog, 2016, for reviews). Prior-task success may lead older adults to be more confident in their cognitive performance. Such increased self-confidence may result in older adults deploying more cognitive resources (e.g., higher processing speed, better executive control) in the target task. This would lead them to be faster via executing strategies more quickly and using the better strategy more often under success condition.

Effects of prior-task success have important consequences for standard practices in the fields of normal and pathological aging, both in research and clinical contexts. The order of tasks administered to young and older adults can dramatically change the picture of aging and pathological effects on human cognition. Finding no negative effects of aging under success conditions contrasts with such negative effects under no-prior task conditions. In other words, magnitudes of age-related changes in a target cognitive domain may be different if these changes are assessed after older adults successfully accomplish a task.

Effects of prior-task success also question the research and clinical practices with specific aging populations (e.g., Alzheimer’s patients) when assessing cognitive functioning. Clinical research may examine whether effects of prior-task success vary with severity of cognitive impairment, and how much patients may benefit from prior-task success. In clinical practice, the order of task administration may lead to underestimation or overestimation of true cognitive capacities in these patients. Neuropsychological tests should be administered in order that maximise participants’ performance if we are to assess spared cognitive resources in patients. Neuropsychologists should also consider that Alzheimer patients may be able to use more efficient strategies and execute each available strategies more efficiently if tested under optimal conditions, where they first successfully accomplish tests before taking harder neuropsychological tests.

## Data Accessibility Statement

Data for this experiment is available at https://osf.io/pkcr6/.

## Footnotes

Given that older adults had higher arithmetic fluency scores than young adults, all analyses have been redone with arithmetic fluency scores entered as covariates. However, the same results came out significant.
